# Practical Implementation of Steroidal and Nonsteroidal Mineralocorticoid Receptor Antagonists in the Management of Heart Failure

**DOI:** 10.1016/j.jacadv.2026.103280

**Published:** 2026-09-25

**Authors:** Erin D. Michos, Andrew P. Ambrosy, Gregg C. Fonarow

**Affiliations:** a Division of Cardiology, Department of Medicine, Johns Hopkins University School of Medicine, Baltimore, Maryland, USA; b Department of Cardiology, Kaiser Permanente San Francisco Medical Center, San Francisco, California, USA; c Division of Research, Kaiser Permanente Northern California, Pleasanton, California, USA; d Division of Cardiology, Department of Medicine, David Geffen School of Medicine at UCLA, Los Angeles, California, USA

**Keywords:** eplerenone, finerenone, mildly reduced ejection fraction, preserved ejection fraction, spironolactone

## Abstract

Mineralocorticoid receptor antagonists (MRAs) are a well-established treatment option for the management of heart failure (HF) with reduced ejection fraction. However, high-quality evidence for the effect of MRAs in the treatment of heart failure with mildly reduced or preserved ejection fraction (HFmrEF/HFpEF) has been limited until recent years. The nonsteroidal MRA finerenone has demonstrated efficacy in this population and was approved for treatment in July 2025 by the U.S. Food and Drug Administration. A call to action is needed to increase the use of guideline-directed medical therapy in HFmrEF/HFpEF with simultaneous or rapid sequence initiation of a combination therapy. This review provides an overview of MRAs approved for use in HF management, with particular focus on HFmrEF/HFpEF. Guideline recommendations and HF label indications are discussed, highlighting practical considerations for the safe use of MRAs in clinical practice. In addition, the therapeutic management of patients with HFmrEF/HFpEF and concomitant cardiovascular, kidney, and metabolic diseases is considered.

## Highlights

• When considered as a single class, MRAs have demonstrated efficacy across the heart failure (HF) spectrum.
• Guideline-directed medical therapy is often underutilized in patients with HF.
• Consider simultaneous or rapid sequence initiation of a combination therapy for patients with HF with mildly reduced or preserved ejection fraction.
• HF management should be optimized and individualized to account for comorbid cardiovascular–kidney–metabolic disorders.

## Evolution of heart failure toward optimized management

Heart failure (HF) is a clinical syndrome characterized by classic symptoms and/or signs caused by a structural or functional defect in the heart together with elevated natriuretic peptide levels and/or evidence of pulmonary or systemic congestion.^1^, ^2^, ^3^ Patients are classified according to left ventricular ejection fraction (LVEF) based on observed variation in prognosis and/or response to treatment. Subgroups include heart failure with reduced ejection fraction (HFrEF) (LVEF ≤40%), heart failure with mildly reduced ejection fraction (HFmrEF; LVEF 41%-49%), and heart failure with preserved ejection fraction (HFpEF) (LVEF ≥50%).^1^^,^^2^ The management approach of HF varies according to these different LVEF categories.^1^^,^^2^^,^^4^

In 2017, an estimated 64.3 million people had HF worldwide,^3^ and the overall prevalence is continuing to rise.^5^ Registry data are variable for the prevalence of HF types according to LVEF, but the overall pattern suggests that HFrEF rates are stable and may be declining, while HFpEF is steadily increasing.^3^ HFpEF now accounts for approximately 50% of all HF cases,^6^ and it is thought that the prevalence of HFmrEF is 10% to 25%.^7^^,^^8^ Factors responsible for the high prevalence of HFpEF include increased life expectancy, a rise in comorbidities (notably obesity)^9^ that increase the risk of HF, and greater clinical recognition of this early stage of disease.^6^ Effective strategies are therefore needed to treat patients with HF across the different EF categories.^10^ Classes of therapy that have been used for the management of HF include the mineralocorticoid receptor antagonists (MRAs), angiotensin receptor–neprilysin inhibitors (ARNis), angiotensin-converting enzyme inhibitors (ACEIs), angiotensin receptor blockers (ARBs), beta-blockers, and sodium–glucose cotransporter-2 inhibitors (SGLT-2is).

This review will cover the different types of MRAs that are approved for use in the management of HF, with a special focus on HFmrEF/HFpEF (Central Illustration).^11^^,^^12^ Guideline and label recommendations for each type of MRA will be discussed, together with practical considerations for their safe use in clinical practice during treatment initiation, titration, and monitoring according to their individual indication labels for HF. Treatment of HFmrEF/HFpEF with MRAs in the wider context of management of inter-related disorders within a cardiovascular-kidney-metabolic (CKM) disease will also be covered.

**Central Illustration:**
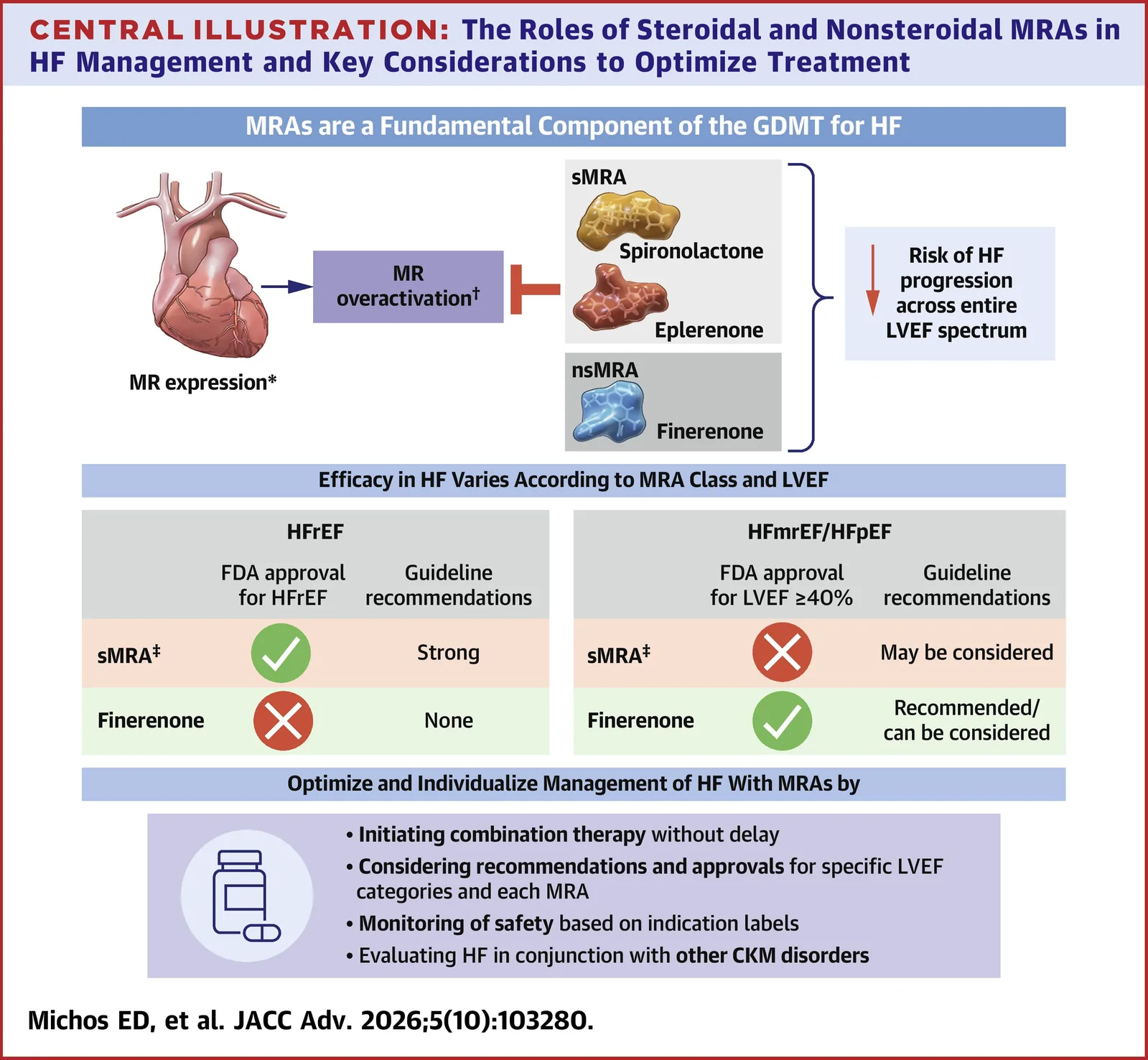
The Roles of Steroidal and Nonsteroidal MRAs in HF Management and Key Considerations to Optimize Treatment ∗The MR is expressed in the heart and blood vessels.^11^^†^Overactivation of the MR in the circulatory system is responsible for adverse cardiovascular effects, such as endothelial dysfunction, vasoconstriction, increased inflammation and oxidative stress, increased thrombosis, atherogenesis, myocardial hypertrophy, apoptosis, and fibrosis.^11^^‡^Spironolactone and eplerenone. CKM = cardiovascular–kidney–metabolic; FDA = U.S. Food and Drug Administration; GDMT = guideline-directed medical therapy; HF = heart failure; HFmrEF = heart failure with mildly reduced ejection fraction; HFpEF = heart failure with preserved ejection fraction; HFrEF = heart failure with reduced ejection fraction; LVEF = left ventricular ejection fraction; MR = mineralocorticoid receptor; MRA = mineralocorticoid receptor antagonist; nsMRA = nonsteroidal MRA; sMRA = steroidal MRA.

### The central role of aldosterone in the neurohormonal hypothesis

The neurohormonal hypothesis was devised to explain mechanisms behind disease progression in HF that could not be attributed to hemodynamic effects.^12^ The hypothesis is that endogenous neurohormonal systems, such as the sympathetic nervous system and the renin–angiotensin–aldosterone system (RAAS), become activated following the initial injury and subsequently cause deleterious effects on the heart and circulation.^12^ Activation of RAAS is now well established as a key pathophysiological feature in HF disease progression.^11^ Aldosterone has therefore become an attractive target for the management of HF as its receptor, the mineralocorticoid receptor (MR), is expressed in the heart and blood vessels. Overactivation of the MR in the circulatory system is responsible for adverse CV effects, such as endothelial dysfunction, vasoconstriction, increased inflammation and oxidative stress, increased thrombosis, atherogenesis, myocardial hypertrophy, apoptosis, and fibrosis.^11^ Key agents for the aldosterone antagonism in HF such as ACEIs, ARBs, and MRAs inhibit this neurohormonal system via different mechanisms, and their combined activity can result in substantial suppression of RAAS.^11^ New agents such as the aldosterone synthase inhibitor vicadrostat, which blocks the synthesis of aldosterone, and the selective MR modulator balcinrenone are also being developed for the treatment of HF.^13^ As these agents are currently investigational, they are outside the scope of this review and will not be covered in detail.

### Overview of the current treatment landscape for HF

The latest guidelines for the management of HFrEF by the American Heart Association/American College of Cardiology/Heart Failure Society of America (AHA/ACC/HFSA) and the European Society of Cardiology (ESC) strongly recommend a quadruple therapy with 4 “pillars” of treatment that include an ARNi/ACEI/ARB, beta-blockers, MRAs, and SGLT-2is.^1^^,^^2^^,^^5^^,^^14^ Combined treatment with these agents offers great potential for improved outcomes in HFrEF as the relative risk reduction (RRR) in mortality estimated using a decision analytical model is over 70% for quadruple therapy vs estimated RRRs of 17% to 35% when agents are administered individually.^15^ Indeed, simultaneous treatment in the STRONG-HF (Safety, Tolerability, and Efficacy of Rapid Optimization, Helped by N-Terminal Pro–Brain Natriuretic Peptide Testing of Heart Failure Therapies) trial that included patients regardless of LVEF (n = 1,078; 67.8% with HFrEF) demonstrated that rapid uptitration of guideline-directed medical therapy (GDMT) including ARNi/ACEI/ARB, beta-blockers, and MRAs after hospital admission for acute HF improved outcomes vs usual care (adjusted risk ratio for the composite endpoint of readmission for HF and all-cause death after 180 days was 0.66; *P* = 0.0021).^16^ It is important to note that SGLT-2is were not administered in the STRONG-HF study because this drug class was not approved for use in HF at study initiation.^16^

By contrast, treatment recommendations for HFmrEF/HFpEF have been less clear than those for HFrEF because of a lack of high-quality evidence for all classes of therapy.^2^ Guidance is urgently needed for early diagnosis and treatment of HFmrEF/HFpEF given its increasing prevalence and the long asymptomatic period that usually precedes an HF diagnosis.^5^ A joint scientific statement from the HFSA and The American Society for Preventive Cardiology (ASPC) has highlighted a continued increase in mortality related to HF despite significant strides in the treatment landscape and a high prevalence of individuals fitting into the “at-risk” (ie, Stage A) or “pre-HF” (ie, Stage B) categories in the general population.^17^ The HFSA/ASPC statement calls for a paradigm shift to prioritize prevention of HF in people at risk of developing HF (primary) and people with an HF diagnosis (secondary).^17^ In line with this scientific statement, effective strategies for the management of HFmrEF/HFpEF and a proactive treatment approach are needed to delay or prevent the onset of HF. More recent guidelines for the management of HFmrEF/HFpEF have started to include stronger recommendations for certain drugs. The focused update of guidelines from the ESC in 2023 included a Class I recommendation for SGLT-2is in HFmrEF/HFpEF following the results from the EMPEROR-Preserved (EMPagliflozin outcomE tRial in Patients With chrOnic heaRt Failure With Preserved Ejection Fraction) and DELIVER (Dapagliflozin Evaluation to Improve the LIVEs of Patients With PReserved Ejection Fraction Heart Failure) trials in patients with LVEF >40% (n = 5,988 and n = 6,263, respectively), although the evidence remained weaker for other drug classes, such as ARNi/ACEI/ARB, MRAs, and beta-blockers.^4^ More data are now beginning to emerge to support the use of GDMT already recommended for HFrEF to be used in HF cases with LVEF >40%. A prespecified analysis of data from STRONG-HF (n = 1,078; 32% with LVEF >40%) revealed that rapid uptitration of GDMT with ARNi/ACEI/ARB, beta-blockers, and MRAs offered the same or greater treatment benefit in patients with HF with LVEF >40% compared with those with HFrEF (composite endpoint of HF rehospitalization or all-cause death at day 180 occurred in 10.7% and 17.4% of patients after rapid uptitration vs 23.3% and 23.7% of patients with usual care in patients with HF with LVEF >40% and HFrEF, respectively).^18^ Furthermore, results from phase 3 trials in HFmrEF/HFpEF have led to new approvals for the ARNi sacubitril/valsartan for patients with an LVEF below normal (<60%)^19^, ^20^, ^21^ and the nonsteroidal MRA (nsMRA) finerenone for patients with LVEF ≥40%.^22^

Despite all the recommendations, GDMTs are underutilized in eligible patients with HFrEF and HFmrEF/HFpEF,^23^ and implementation of these therapies remains an unmet need in clinical practice. This appears to be particularly true for MRAs compared with other evidence-based therapies for HF^24^ because of concerns about side effects such as hyperkalemia, hypotension, and worsening kidney function, particularly in older patients.^25^^,^^26^ The monitoring burden associated with the use of MRAs may also reduce uptake, and guidelines for laboratory monitoring of MRAs appear to be followed infrequently in clinical practice.^27^

With the aim of overcoming clinical inertia with GDMT, Greene et al^28^ proposed that treatment with all 4 foundational therapies should be initiated rapidly during hospitalization for HFrEF, even if treatment begins at a low dose initially for all agents. Minimizing time to quadruple therapy is the highest therapeutic priority for HFrEF given that delaying the initiation of one or more of these treatments in eligible patients contributes to excess clinical risk. Once all 4 therapies have been initiated, medication doses can be escalated to their individual target doses (as tolerated) within 4 to 6 weeks.^28^ Similarly, it is also proposed that treatment for HFmrEF/HFpEF should involve simultaneous or rapid sequence initiation of the combination therapy without delay.^29^ However, therapeutic inertia is likely to be prominent in HFmrEF/HFpEF given the historical lack of efficacious treatments for these patients.^30^ It should be recognized that there is great potential for synergistic effects with the new drug classes available in HFmrEF/HFpEF because of nonoverlapping therapeutic targets and their ability to impact distinct metabolic, hemodynamic, and inflammatory pathways involved in disease progression.^31^, ^32^, ^33^ Another potential barrier for the use of new drugs in the management of HFmrEF/HFpEF is cost.^34^ The absence of robust cost-effectiveness analyses for these agents may restrict their use, particularly in areas where resources are limited. In addition, other general barriers such as knowledge/awareness gaps and bias can hinder implementation of these treatment approaches to optimize management of HF for all patients.^35^ Strategies to encourage implementation need to be adopted at all stages of patient care; for example, development of protocols for rapid initiation and titration of GDMT, formation of virtual GDMT-optimization teams (including nonphysician care providers such as pharmacists and nurses), and utilization of electronic health record alerts in the hospital; better HF education for the patient and the health care provider at discharge; digital innovations in the outpatient setting such as mobile applications and remote monitoring; and audit and feedback of current metrics to drive improvement.^35^^,^^36^

## Introduction to steroidal MRAs and nsMRAs approved for the treatment of HF

There are 2 different classes of MRA: steroidal mineralocorticoid receptor antagonists (sMRAs) (the first-generation MRA spironolactone and the second-generation MRA eplerenone) and nsMRAs (including the third-generation MRA finerenone).^37^^,^^38^ Both classes of MRA inhibit the MR but have different pharmacokinetic and pharmacodynamic properties that can lead to differences in their clinical effects.^38^ The nsMRAs have been designed to have a bulky structure vs sMRAs, which alter selectivity for the MR and other steroid hormone receptors.^38^ The nsMRA finerenone has better specificity for the MR, so it inhibits the MR with greater potency than spironolactone or eplerenone (MR half-maximal inhibitory concentration of 18 nM vs 24 nM and 990 nM, respectively).^37^, ^38^, ^39^ As a result, the off-target effects on androgen and progesterone receptors that occur with sMRAs are not observed with nsMRAs. These off-target effects can potentially lead to the development of gynecomastia and hormonal imbalances that may limit sMRA use.^37^

As with SGLT-2is, which have demonstrated efficacy in HF regardless of EF, MRAs have also been shown to be efficacious across the entire LVEF spectrum when considered as a single therapeutic class.^1^^,^^4^^,^^40^ However, each type of MRA is restricted to approval in a specific HF population. The sMRA spironolactone has long been approved by the U.S. Food and Drug Administration (FDA) and the European Medicines Agency (EMA) for use in HFrEF.^41^^,^^42^ Approvals by the FDA and the EMA subsequently followed in HFrEF for the sMRA eplerenone.^43^^,^^44^ In July 2025, the nsMRA finerenone was approved by the FDA for the treatment of HFmrEF/HFpEF,^22^ and in March 2026, the EMA also approved finerenone for the same indication.^45^^,^^46^ A summary of the FDA indications for spironolactone, eplerenone, and finerenone in HF is shown in Table 1.^22^^,^^41^^,^^43^^,^^47^, ^48^, ^49^, ^50^ Finerenone is also approved by the FDA and the EMA for patients with chronic kidney disease (CKD) and type 2 diabetes (T2D) to reduce the risk of sustained estimated glomerular filtration rate (eGFR) decline, end-stage kidney disease, CV death, nonfatal myocardial infarction (MI), and hospitalization for HF (HHF).^22^

**Table 1:** FDA-Approved Indications for MRAs in HF

Agent	Approval Date in HF	HFrEF Indication	HFmrEF/HFpEF Indication
Spironolactone	1999^47^	The treatment of NYHA class III–IV HF and reduced ejection fraction to increase survival, manage edema, and to reduce the need for HHF^41^	Not currently approved. Spironolactone is currently being investigated in HFmrEF/HFpEF in the SPIRRIT-HFpEF trial^48^
Eplerenone	2003^49^	Improving survival of stable adult patients with symptomatic HF with reduced ejection fraction after an acute MI^43^	Not currently approved
Finerenone	2025^22^	Not currently approved. Finerenone is currently being investigated in HFrEF in the FINALITY-HF trial^50^	CV death, HHF, and urgent HF visits in adult patients with HF with LVEF ≥40%^22^

CV = cardiovascular; FDA = U.S. Food and Drug Administration; HF = heart failure; HFmrEF = heart failure with mildly reduced ejection fraction; HFpEF = heart failure with preserved ejection fraction; HFrEF = heart failure with reduced ejection fraction; HHF = hospitalization for heart failure; LVEF = left ventricular ejection fraction; MI = myocardial infarction; MRA = mineralocorticoid receptor antagonists.

### Indications and guideline recommendations for sMRAs and nsMRAs

#### Steroidal MRAs

Spironolactone has been used for the treatment of HFrEF for more than 2 decades following results from the landmark RALES (Randomized Aldactone Evaluation Study) trial (N = 1,663) in 1999 that led to FDA approval for its use in patients with HFrEF and NYHA class III-IV symptoms.^41^^,^^51^ Eplerenone was subsequently approved by the FDA in 2003 based on data from the EPHESUS (Eplerenone Post–Acute Myocardial Infarction Heart Failure Efficacy and Survival Study, N = 6,632) for HFrEF after an acute MI.^49^^,^^52^ EMPHASIS-HF (Eplerenone in Mild Patients Hospitalization and Survival Study in Heart Failure, N = 2,737) that followed expanded the use of eplerenone for HFrEF in patients with NYHA functional class II symptoms.^53^

However, spironolactone and eplerenone are not currently approved by the FDA for treatment of HF in patients with LVEF ≥40% (HFmrEF/HFpEF). Although sMRAs may be used for the management of these patients in clinical practice, the evidence for a demonstrated benefit in clinical trials is limited. The TOPCAT (Aldosterone Antagonist Therapy for Adults With Heart Failure and Preserved Systolic Function, N = 3,445) trial was designed to assess the efficacy of spironolactone in HFmrEF/HFpEF (LVEF range 44%-85%) vs placebo.^54^^,^^55^ The primary composite endpoint of death from CV causes, aborted cardiac arrest, or HHF was not met (*P* = 0.14).^54^ A retrospective analysis from the TOPCAT study showed that LVEF modified the treatment effect of spironolactone for the primary outcome (*P* = 0.046) and HHF (*P* = 0.039). The benefit of spironolactone was estimated to be stronger in patients with HF with LVEF <50% vs LVEF ≥60%.^55^ However, results from TOPCAT were confounded by several factors, which makes interpretation difficult.^56^ A post hoc analysis revealed that a significant difference was observed for the primary outcome between the placebo groups for the Americas (United States, Canada, Brazil, and Argentina) vs Eastern Europe (Russia and Georgia; *P* < 0.001), and the hazard ratio for spironolactone treatment in the Americas was 0.82 (95% CI: 0.69-0.98) compared with 1.10 (95% CI: 0.79-1.51) in Eastern Europe.^56^ SPIRIT-HF (Spironolactone in the Treatment of Heart Failure—a Double-blind, Randomized, Placebo-controlled, Parallel Group Interventional Phase III Study to Determine Efficacy and Safety of Spironolactone on the Composite Endpoint of Recurrent Heart Failure Hospitalizations and Cardiovascular Death in Patients with Heart Failure with Mid-range or Preserved Ejection Fraction, N = 743) was subsequently designed to further investigate the efficacy of spironolactone in HFmrEF/HFpEF but was terminated in 2025 because of the lack of funding and access to study drugs.^57^ A high rate of discontinuation occurred in the SPIRIT-HF trial possibly because of the COVID-19 pandemic (trial was conducted during 2018-2024) and/or side effects of spironolactone. Consequently, statistical power was reduced in the study with a 65% likelihood of a type II error, making it difficult to draw meaningful conclusions^58^^,^^59^; the primary endpoint was not met, and safety concerns including higher rates of total hospitalizations, hypotension, hyperkalemia, and kidney events were reported with spironolactone vs placebo.^58^^,^^59^ Importantly, the only data that reached statistical significance were total all-cause hospitalizations through 2 years, which were more common with spironolactone vs placebo (rate ratio: 1.40; 95% CI: 1.07-1.83).^58^ Furthermore, a meta-analysis of pooled data from the SPIRIT-HF and the TOPCAT Americas cohorts showed no significant effect on outcomes with spironolactone.^59^ At this time, it is difficult to interpret the data from SPIRIT-HF as the neutral findings in the trial may have been due to poor tolerability of spironolactone in this patient population or potentially due to aldosterone reactivation following spironolactone discontinuation.^60^ Looking ahead, the current uncertainty surrounding spironolactone in HFmrEF/HFpEF may be resolved by results from the ongoing SPIRRIT-HFpEF (Spironolactone Initiation Registry Randomized Interventional Trial in Heart Failure with Preserved Ejection Fraction) registry–based study (estimated enrollment N = 2,000).^48^^,^^61^ In contrast to spironolactone, data are extremely limited for eplerenone in HFmrEF/HFpEF, and any guideline recommendations that include this sMRA appear to be based on observations with spironolactone.^1^^,^^2^^,^^62^^,^^63^

Based on the limited evidence base, guideline recommendations are variable for sMRA, with use in HFmrEF/HFpEF mostly being suggested or considered based mainly on data from the TOPCAT trial (Table 2).^1^^,^^2^^,^^4^^,^^10^^,^^22^^,^^41^^,^^43^^,^^48^^,^^50^^,^^62^, ^63^, ^64^, ^65^, ^66^, ^67^ ESC and AHA/ACC/HFSA guidelines state that treatment with an sMRA may be considered in patients with HFmrEF (Class IIb), and the Japanese Circulation Society/Japanese Heart Failure Society (JCS/JHFS) guidelines offer the same recommendation for symptomatic HFmrEF (Class IIb).^1^^,^^2^^,^^62^ In cases of HFpEF, an sMRA may be considered in selected patients (AHA/ACC/HFSA, Class IIb), in symptomatic patients (JCS/JHFS, Class IIb), or used with an SGLT-2i (ACC Expert Consensus, Class IIb).^2^^,^^62^^,^^63^ The iCARDIO Alliance Global Implementation Guidelines suggest the use of spironolactone, but not eplerenone, in HFpEF.^10^ Although eplerenone has the same mechanism of action as spironolactone, its exclusion may be due to a lack of dedicated trial data in this specific patient population or because spironolactone demonstrates comparable efficacy at a substantially lower cost in HF.^68^

**Table 2:** Guideline Recommendations for sMRAs and nsMRAs

	sMRA	Finerenone
HFmrEF/HFpEF	**Not currently approved** Spironolactone is currently being investigated in HFmrEF/HFpEF in the SPIRRIT-HFpEF trial^48^	**FDA approved**^22^
ESC, 2021^1^^,^^a^	**May be considered** in patients with HFmrEF (**Class IIb**) to reduce the risk of HHF and death.	–
AHA/ACC/HFSA, 2022^2^^,^^a^	**May be considered** in patients with HFmrEF (**Class IIb**) to reduce the risk of HHF and death and in selected patients with HFpEF (**Class IIb**) to reduce hospitalizations.	–
ACC Expert Consensus, 2023^63^^,^^a^	**Use with an SGLT-2i** in HFpEF **(Class IIb)**	–
iCARDIO Alliance, 2025^10^	**Spironolactone is suggested** for use in HFpEF	**Recommended** for HFpEF
HFAI, 2025^64^	–	Finerenone **can be considered** in patients with HFmrEF/HFpEF and an eGFR >25 mL/min/1.73 m^2^
JCS/JHFS, 2025^62^	**May be considered** to reduce the risk of CV death or HHF in symptomatic HFmrEF (**Class IIb**) and to reduce HHF in symptomatic HFpEF (**Class IIb**)	**Can be considered** for symptomatic HFmrEF/HFpEF to reduce the risk of CV death or HF exacerbation events (**Class IIa**)
NICE, 2025^65^	**Consider an MRA** for treating HFmrEF/HFpEF	
CCS/CHFS, 2025^66^	**MRAs are recommended** for people with symptomatic HF and LVEF >40% to reduce the risk of HHF (**strong recommendation**)	
HFrEF	**FDA approved**^41^^,^^43^	**Not currently approved.** Finerenone is currently being investigated in HFrEF in the FINALITY-HF trial^50^
ESC, 2021^1^^,^^a^	**Recommended** to reduce the risk of HHF and death (**Class Ia**)	–
AHA/ACC/HFSA, 2022^2^^,^^a^	**Recommended** to reduce morbidity and mortality if eGFR is >30 mL/min/1.73 m^2^ and serum potassium is <5.0 mEq/L.^b^ Careful monitoring of potassium, kidney function, and diuretic dosing should be performed at initiation and closely monitored thereafter to minimize risk of hyperkalemia and renal insufficiency (**class Ia**)	–
HFAI, 2025^64^	MRA (spironolactone and eplerenone) are a **foundational pillar of treatment for HFrEF**	–
iCARDIO Alliance, 2025^10^	**Strongly recommended** to reduce morbidity and mortality if eGFR is >30 mL/min/1.73 m^2^ and serum potassium is <5.0 mEq/L	–
JCS/JHFS, 2025^62^	**Recommended** to reduce morbidity and mortality (**Class Ia**)^b^	–
NICE, 2025^65^	Offer an ACEI, a beta-blocker, **an MRA**, and an SGLT-2i	
HF in CKD and T2D	–	**FDA approved**^22^
ESC, 2023^4^	–	**Recommended** to reduce the risk of HHF (**Class Ia**)
JCS/JHFS, 2025^62^	–	**Should be used** to prevent the onset of HF (**Class Ia**)
CCS/CHFS^66^		Finerenone **may be prioritized as the MRA of choice**, if accessible and affordable, because there is high-quality evidence to support the use of this agent in improving CV and kidney outcomes
CKD with T2D	**–**	**FDA approved**^22^
KDIGO, 2022^67^	–	**Suggest an nsMRA with proven kidney or CV benefit** for patients with T2D, an eGFR ≥25 mL/min per 1.73 m^2^, normal serum potassium concentration, and albuminuria (≥30 mg/g) despite maximum tolerated dose of RASi (**Class IIa**)

Text in bold denotes key information.

ACC = American College of Cardiology; ACEI = angiotensin-converting enzyme inhibitor; AHA = American Heart Association; CCS = Canadian Cardiovascular Society; CHFS = Canadian Heart Failure Society; CKD = chronic kidney disease; CV = cardiovascular; eGFR = estimated glomerular filtration rate; ESC = European Society of Cardiology; FDA = U.S. Food and Drug Administration; FINEARTS-HF = FINerenone trial to investigate Efficacy and sAfety superior to placebo in paTientS with Heart Failure; HF = heart failure; HFAI = Heart Failure Association of India; HFmrEF/HFpEF = HF with mildly reduced or preserved ejection fraction; HFrEF = heart failure with reduced ejection fraction; HFSA = Heart Failure Society of America; HHF = hospitalization for heart failure; JCS = Japanese Circulation Society; JHFS = Japanese Heart Failure Society; KDIGO = Kidney Disease: Improving Global Outcomes; MRA = mineralocorticoid receptor antagonist; NICE = National Institute for Health and Care Excellence; nsMRA = nonsteroidal mineralocorticoid receptor antagonist; RASi = renin–angiotensin system inhibitor; SGLT-2i = sodium–glucose cotransporter-2 inhibitor; sMRA = steroidal mineralocorticoid receptor antagonist; T2D = type 2 diabetes.

a Published prior to the availability of data from FINEARTS-HF and subsequent FDA approval.

b In patients with HFrEF and NYHA functional class II-IV HF.

#### Nonsteroidal MRA

The nsMRA finerenone was originally approved by the FDA in 2021 for reducing the risk of CV and kidney events in patients with CKD and T2D, and Kidney Disease: Improving Global Outcomes clinical practice guidelines suggest the use of finerenone in these patients.^67^ Since July 2025, finerenone is also indicated for the treatment of HF with LVEF ≥40%.^22^ Finerenone is the only MRA currently indicated by the FDA for use in HFmrEF/HFpEF. Approval for its use in HFmrEF/HFpEF by the FDA was granted based on the FINEARTS-HF (FINerenone trial to investigate Efficacy and sAfety superior to placebo in paTientS with Heart Failure, N = 6,001), which showed that finerenone reduced the rate of a composite clinical outcome of worsening HF events and death from CV causes compared with placebo in patients with HFmrEF/HFpEF (risk ratio: 0.84; 95% CI: 0.74-0.95; *P* = 0.007).^22^^,^^40^ The reduced risk was consistent across a wide range of prespecified subgroups, including but not limited to, age; sex; race; LVEF (<60% or ≥60%); ACEI, ARB, or ARNi use at baseline; SGLT-2i use at baseline; and eGFR at baseline (<60 mL/min/1.73 m^2^ or ≥60 mL/min/1.73 m^2^).^40^ A pooled analysis of patients with CKM syndrome has provided further evidence of the effect of finerenone on CV outcomes.^69^ FINE-HEART (An Integrated Pooled Analysis of Finerenone across 3 Phase III Trials of Heart Failure and Chronic Kidney Disease and Type 2 Diabetes; N = 18,991; 36.9% of patients with a history of HF at baseline) included patient data from FINEARTS-HF on HFmrEF/HFpEF together with 2 trials on CKD with T2D, FIDELIO-DKD (FInerenone in reducing kiDnEy faiLure and dIsease prOgression in Diabetic Kidney Disease) and FIGARO-DKD (FInerenone in reducinG cArdiovascular moRtality and mOrbidity in Diabetic Kidney Disease). Data from FIDELIO-DKD and FIGARO-DKD provided the basis for the indication of finerenone use in CKD and T2D approved by the FDA.^22^ The REDEFINE-HF (Efficacy and Safety of Finerenone on Morbidity and Mortality Among Heart Failure Patients With Left Ventricular Ejection Fraction Greater Than or Equal to 40% Hospitalized Due to an Episode of Acute Decompensated Heart Failure) trial with cases of LVEF ≥40% (N = 5,200),^70^ the CONFIRMATION-HF (Combined Efficacy and Safety of an Early, Intensive, Management Strategy with Finerenone and SGLT2 Inhibitor in Patients Hospitalized with Heart Failure, N = 1,500) study in patients with any LVEF,^37^^,^^71^ and the FINALITY-HF (A Randomized, Double-blind, Placebo-controlled Pragmatic Study to Evaluate Finerenone on Clinical Efficacy and Safety in Patients with Heart Failure and Reduced Ejection Fraction Who Are Intolerant of or Not Eligible for Treatment with Steroidal Mineralocorticoid Receptor Antagonists) trial on HFrEF (N = 2,600)^50^ are ongoing studies that will provide further evidence for the safety and efficacy of finerenone in HF.

The latest guidelines from AHA/ACC/HFSA and ESC for the treatment of HF were published before the completion of FINEARTS-HF and subsequent approval of finerenone use in HFmrEF/HFpEF.^2^^,^^4^^,^^40^ However, specific recommendations for finerenone use in HF with LVEF ≥40% are now beginning to appear in more recent guidelines published in 2025 (Table 2). The iCARDIO Alliance recommends finerenone for the management of HFpEF,^10^ and JCS/JHFS state that finerenone should be considered for symptomatic HFmrEF/HFpEF to reduce the risk of CV death or HF exacerbation events (Class IIa).^62^ The Heart Failure Association of India guidelines note that finerenone can be considered in patients with HFmrEF/HFpEF and an eGFR >25 mL/min/1.73 m^2^.^64^ Multiple expert opinion articles published in 2025 also recommend concomitant treatment with finerenone and an SGLT-2i for HFmrEF/HFpEF.^29^^,^^72^^,^^73^ Guidelines from the National Institute for Health and Care Excellence in the UK and the Canadian Cardiovascular Society/Canadian Heart Failure Society (CCS/CHFS) do not specify which class of MRA should be used in their recommendations for HFmrEF/HFpEF (Table 2).^65^^,^^66^ The CCS/CHFS recommends MRAs for people with symptomatic HF and LVEF >40% to reduce the risk of HHF but notes that there are no head-to-head efficacy data or cost-effective analyses that can be used to distinguish between sMRA and nsMRA, and that sMRAs are widely accessible, generally affordable and already used routinely for the management of HFrEF.^66^ These factors highlighted by the CCS/CHFS may influence overall preference for one MRA over another for the management of HFmrEF/HFpEF until further data are available.

## Practical considerations for the use of sMRAs and nsMRAs approved for HF

### Initiation and titration of treatment based on indication labels for HF

#### sMRAs (approved in HFrEF)

Given that treatment with spironolactone and eplerenone can cause hyperkalemia and that worsening kidney function may also occur with spironolactone,^41^^,^^43^ key practice points need to be followed to ensure the safe use of sMRAs (Figure 1). Before treatment initiation, serum potassium levels need to be assessed for both spironolactone and eplerenone, and kidney function also needs to be evaluated to determine whether spironolactone is an appropriate treatment option (Figures 1A and 1C).

**Figure 1:**
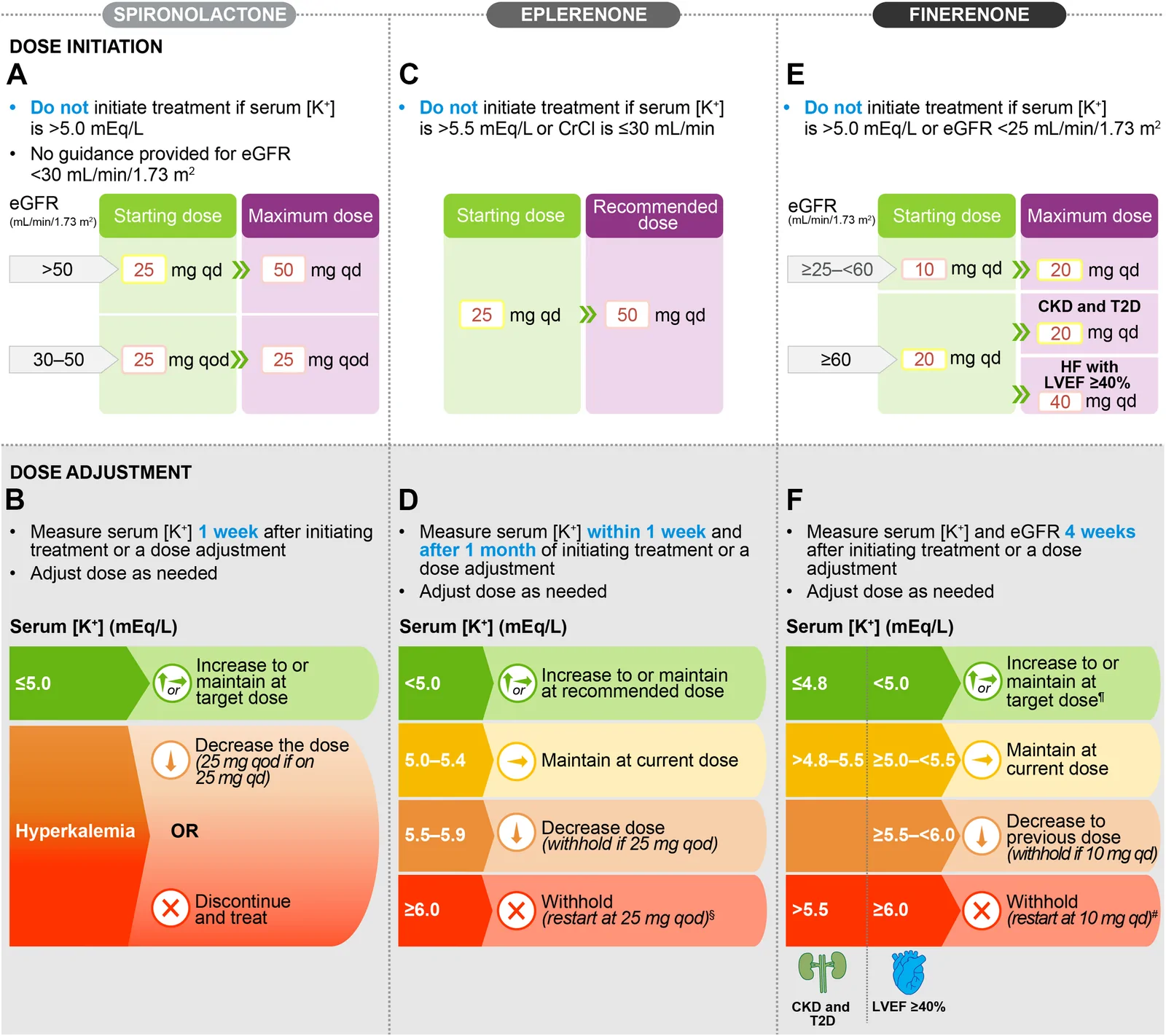
Initiation and Dose Adjustment Protocols for Spironolactone,∗ Eplerenone,^†^ and Finerenone^‡,^^22^^,^^41^^,^^43^ ∗Approved by the FDA for HFrEF, hypertension, edema, and primary hyperaldosteronism; ^†^approved by the FDA for HFrEF after acute myocardial infarction and hypertension; ^‡^approved by the FDA for HF with LVEF ≥40% and CKD with T2D; ^§^when potassium levels are <5.5 mEq/L; ^¶^for CKD with T2D, the target daily dose is 20 mg; the dose can be increased from 10 mg qd if eGFR has decreased by ≤30%. For HF with LVEF ≥40%, the target daily dose is 20 mg or 40 mg if eGFR at initiation is ≥25 to <60 mL/min/1.73 m^2^ or ≥60 mL/min/1.73 m^2^, respectively; dose can be increased to the target dose provided eGFR has decreased by ≤30%; ^#^for CKD with T2D, consider restarting at 10 mg qd when serum [K^+^] ≤5.0 mEq/L. For HF with LVEF ≥40%, if repeated serum [K^+^] measurements are ≥5.5 mEq/L, restart at 10 mg qd when serum [K^+^] <5.0 mEq/L. CKD = chronic kidney disease; CrCl = creatinine clearance; eGFR = estimated glomerular filtration rate; FDA = U.S. Food and Drug Administration; HF, heart failure; HFrEF = heart failure with reduced ejection fraction; [K^+^] = potassium concentration; LVEF = left ventricular ejection fraction; qd = once daily; qod = every other day; T2D = type 2 diabetes.

Treatment with an sMRA for HFrEF should not be initiated if serum potassium is >5.0 mEq/L for spironolactone (Figure 1A) or >5.5 mEq/L for eplerenone (Figure 1C).^41^^,^^43^ Kidney function also needs to be determined before treatment with spironolactone to establish the appropriate starting dose (Figure 1A).^41^ For spironolactone, people with HFrEF and an eGFR >50 mL/min/1.73 m^2^ should begin treatment with 25 mg once daily, but the dose should be reduced to 25 mg every other day (although 12.5 mg daily is more commonly prescribed in clinical practice) in people with an eGFR of 30 to 50 mL/min/1.73 m^2^.^41^ By contrast, eGFR levels do not need to be measured with eplerenone, and all patients with HFrEF should begin treatment with 25 mg once daily (Figure 1C).^43^ Following the initiation of treatment for HFrEF, the daily dose of spironolactone may be increased to 50 mg if 25 mg is well tolerated, and a dose increase is clinically indicated (Figure 1A).^41^ Eplerenone should also be uptitrated from 25 mg to 50 mg once daily if tolerated in cases of HFrEF post MI (Figure 1C).^43^

#### sMRAs (limited evidence for use in patients with HFmrEF/HFpEF)

Although spironolactone is not specifically indicated for HF with LVEF ≥40%, it is assumed that the dose administered to patients with HFmrEF/HFpEF is similar to the dose approved for HFrEF. Guidelines do not mention a specific dose of spironolactone for HFmrEF/HFpEF, but recommendations that include supportive evidence refer to data from the TOPCAT study on HFmrEF/HFpEF.^1^^,^^2^^,^^62^ The dose of spironolactone approved for HFrEF is based on data from the RALES trial (N = 1,663) that investigated a starting dose of 25 mg of spironolactone once daily, which was uptitrated to 50 mg once daily.^51^ The TOPCAT study on HFmrEF/HFpEF subsequently evaluated a spironolactone starting dose of 15 mg once daily that was uptitrated to a maximum of 45 mg within the first 4 months of treatment.^54^ Although the primary endpoint was not reached in the TOPCAT study, the mean dose of spironolactone at 8 months was similar to the mean dose that was administered in RALES (25 mg vs 26 mg daily, respectively), supporting the approved dose schedule in HFrEF.^51^^,^^54^

Similarly, eplerenone is not indicated for HF with LVEF ≥40%, but it is assumed that the dose administered to patients with HFmrEF/HFpEF is similar to the dose approved for HFrEF. Guidelines that recommend an sMRA for HFmrEF/HFpEF do not mention any supportive evidence specifically for eplerenone.^1^^,^^2^^,^^62^ Data from the EPHESUS trial (N = 6,632) of patients with HF with LVEF ≤40% after an acute MI formed the basis of the FDA approval for eplerenone in HFrEF.^43^^,^^52^ A starting dose of 25 mg once daily that was uptitrated to 50 mg once daily after 4 weeks was used in the EPHESUS study. Efficacy of this dose was confirmed in a subsequent trial, EMPHASIS-HF (N = 2,737), of patients with LVEF ≤30% (or >30 to ≤35 with a QRS duration of >130 msec on electrocardiography) and mild symptoms (NYHA functional class II).^53^

#### nsMRA (approved for HFmrEF/HFpEF)

Hyperkalemia and worsening kidney function may also occur with finerenone treatment, so similar key practice points to those recommended for sMRAs should be followed to ensure the safe use of finerenone.^22^ The dose of finerenone should also be managed appropriately, especially for patients with CKD. Given that finerenone is also approved for CKD in patients with T2D, additional points need to be considered for patients with HFmrEF/HFpEF and CKD (discussed in detail in the Treatment of HFmrEF/HFpEF with comorbid CKD section).

Treatment should not be initiated with finerenone if serum potassium is >5.0 mEq/L (Figure 1E).^22^ Kidney function also needs to be determined before finerenone is administered. Patients with an eGFR of ≥60 mL/min/1.73 m^2^ can begin treatment with 20 mg of finerenone, whereas patients with an eGFR of ≥25 to <60 mL/min/1.73 m^2^ should start with 10 mg of finerenone. Finerenone initiation is not recommended in patients who have an eGFR of <25 mL/min/1.73 m^2^ (Figure 1E).^22^ The target dose of finerenone depends on the primary indication for treatment (HFmrEF/HFpEF or CKD with T2D) (Figure 1E, Table 3). The maximum recommended dose of finerenone in patients with HFmrEF/HFpEF is 40 mg once daily, but the target dose for an individual depends on their kidney function at treatment initiation.^22^ The target daily dose is 40 mg if eGFR is ≥60 mL/min/1.73 m^2^ and 20 mg if eGFR is ≥25 to <60 mL/min/1.73 m^2^ (Figures 1E and 2).

**Table 3:** Comparison of Finerenone Indications for CKD With T2D and HF With LVEF ≥40%^22^

	CKD in T2D	HF With LVEF ≥40%
Indicated to reduce the risk of	• CV death • HHF	
	• Sustained eGFR decline • End-stage kidney disease • Nonfatal MI	• Urgent HF visits
Prior to administration	• Measure serum potassium levels and eGFR • Do not initiate if serum potassium is >5.0 mEq/L	
Recommended once daily starting dose of finerenone (mg) based on eGFR (mL/min/1.73 m^2^)		
≥60	20	
≥25 to <60	10	
<25	Not recommended	
Target once daily dose of finerenone	20 mg	40 mg if eGFR ≥60 20 mg if eGFR ≥25 to <60
Dose adjustment categories (based on serum potassium mEq/L)		
Increase	• ≤4.8 for 10 mg (maintain for 20 mg)	• <5.0 for 10 mg and 20 mg (maintain for 20 mg if eGFR <60 at initiation and 40 mg)
Maintain	• >4.8 to 5.5	• ≥5.0 to <5.5
Withhold	• >5.5	• ≥5.5 to <6.0 mEq/L for 10-mg dose (reduce dose for 20 mg and 40 mg) - ≥6.0 mEq/L in all cases
Restart	• ≤5.0 (restart with 10 mg)	• <5.5 (restart with 10 mg)
Warnings and precautions	• Hyperkalemia	
		• Worsening kidney function

Abbreviations as in Tables 1 and 2.

**Figure 2:**
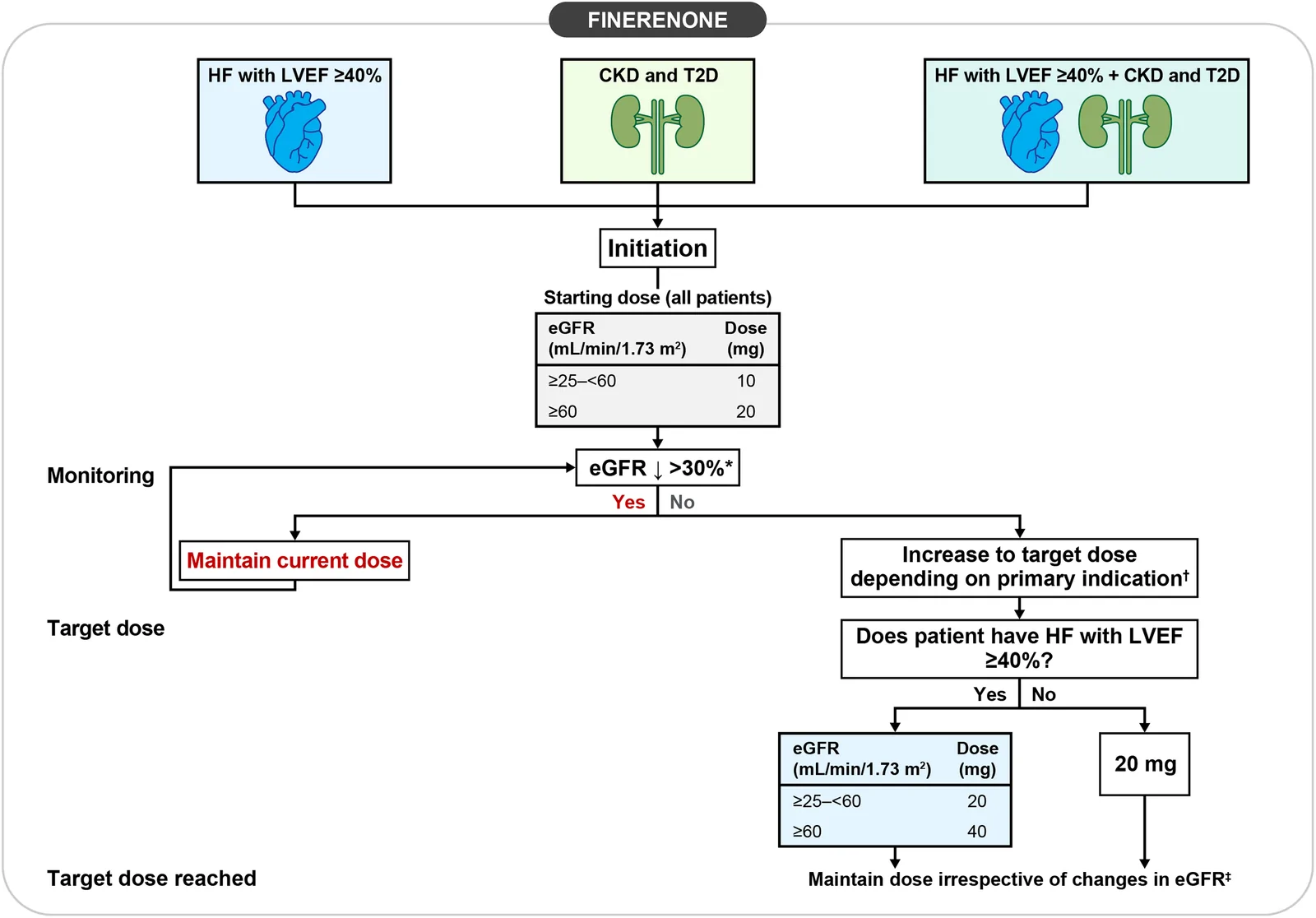
Finerenone Dosing for Patients With HF With LVEF ≥40%, CKD and T2D, or HF With LVEF ≥40% Plus CKD and T2D ∗Compared with previous measurement and provided serum potassium levels are ≤4.8 mEq/L for a primary indication of CKD and T2D or <5.0 mEq/L for a primary indication of HF with LVEF ≥40%. The dose adjustment schedule should be followed based on serum potassium levels if these thresholds are exceeded (Figure 1F). ^†^The target dose should be selected based on the primary indication. In the absence of a specific label or guideline direction on dose selection, when both indications coexist, we suggest that for patients who meet the HF with mildly reduced or preserved ejection fraction indication, the HF indication should be regarded as the primary indication. ^‡^Measure eGFR periodically during maintenance treatment in patients with HF. Consider delaying uptitration or interrupting treatment with finerenone in patients with HF who develop clinically significant worsening of kidney function. HFpEF = heart failure with preserved ejection fraction; other abbreviations as in Figure 1.

An early study of finerenone in patients with HFrEF and CKD revealed that a finerenone dose of 5 to 10 mg/d was as effective as spironolactone doses of 25 to 50 mg/d in terms of reducing levels of B-type natriuretic peptide (BNP), N-terminal proBNP, and albuminuria (part B of the ARTS [MinerAlocorticoid Receptor antagonist Tolerability Study] trial, N = 392).^74^ The appropriate dose of finerenone for use in HF was first investigated in the ARTS-HF (Miner Alocorticoid Receptor antagonist Tolerability Study-Heart Failure) phase 2b trial that evaluated a variety of finerenone doses vs eplerenone in patients with worsening chronic HFrEF with CKD and/or T2D (N = 1,066).^75^ A finerenone starting dose of 10 mg per day that was uptitrated to 20 mg/d after 30 days of treatment reduced levels of N-terminal proBNP to a similar extent as 25 to 50 mg of eplerenone per day and resulted in the greatest reduction in a composite clinical outcome (death from any cause, CV hospitalization, or emergency presentation for worsening chronic HF until day 90) with an acceptable safety profile. Thus, the finerenone dosing schedule was shown to be equivalent to 25 to 50 mg of eplerenone over a period of 3 months in the ARTS-HF trial.^75^

Subsequent phase 3 studies of finerenone have demonstrated the safety of this dosing schedule (10 mg/d uptitrated to 20 mg/d) in patients with CKD and T2D on top of a maximum tolerated dose of ACEi or ARB (FIDELIO-DKD and FIGARO-DKD).^76^^,^^77^ More recently, the FINEARTS-HF phase 3 trial (N = 6,001) showed the efficacy and safety of a higher finerenone target daily dose of 40 mg in participants with HFmrEF/HFpEF who had an eGFR of ≥60 mL/min/1.73 m^2^ and who were uptitrated from a starting dose of 20 mg.^40^^,^^78^ The higher dose of finerenone in FINEARTS-HF was selected based on phase 2 results from ARTS-HF, which revealed that treatment effects on natriuretic peptide levels were dose dependent.^75^^,^^78^

### Monitoring and dose adjustments based on indication labels for HF

Following the initiation of treatment with an MRA, all patients should be monitored to reduce the risk of hyperkalemia.^22^^,^^41^^,^^43^ However, inadequate monitoring of serum potassium levels is a common problem and remains suboptimal in the postinitiation phase despite regular monitoring being recommended in clinical guidelines.^27^^,^^79^^,^^80^ Monitoring requirements vary between the different sMRAs and the nsMRA finerenone, so the guidance provided in the product label needs to be carefully followed (Figures 1B, 1D, 1F, and 3).

**Figure 3:**
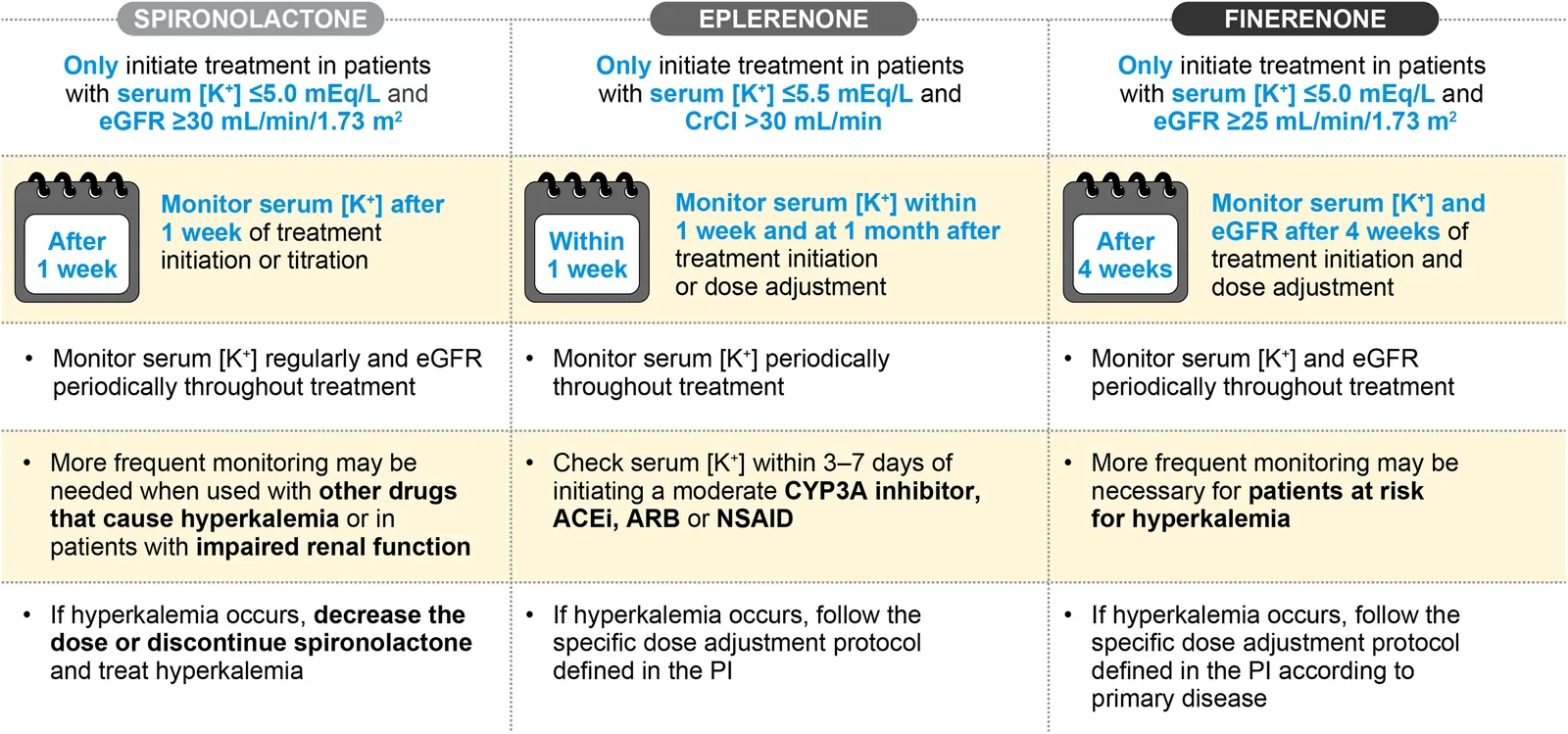
Serum Potassium Monitoring Requirements for Spironolactone,∗ Eplerenone,^†^ and Finerenone^‡,^^22^^,^^41^^,^^43^ ∗Spironolactone is approved by the FDA for HFrEF, hypertension, edema, and primary hyperaldosteronism. ^†^Eplerenone is approved by the FDA for HFrEF after acute myocardial infarction and hypertension. ^‡^Finerenone is approved by the FDA for patients with HF with ≥40% LVEF and patients with CKD and T2D. ACEi = angiotensin-converting enzyme inhibitor; ARB = angiotensin receptor blocker; CYP3A = cytochrome P450, family 3, subfamily A; NSAID = nonsteroidal anti-inflammatory drug; PI = prescribing information; other abbreviations as in Figure 1.

For both sMRAs and nsMRAs, more frequent monitoring may be necessary for patients at risk of hyperkalemia, including those on concomitant medications that interfere with potassium homeostasis or patients with impaired renal function (Figure 3).^22^^,^^41^^,^^43^ The CARE-HK registry that included 38.7% of patients with HFmrEF/HFpEF (N = 2,558) investigated outcomes in patients at high risk of hyperkalemia across the spectrum of HF. A total of 29.2% patients in the registry experienced a hyperkalemic event during treatment, and of these patients, only 3.6% had their RAAS inhibitors or MRA treatment downtitrated or discontinued within 30 days.^81^ These data highlight the importance of frequent potassium monitoring for patients receiving treatment for HF, although, in many cases, treatment modification is not required as the hyperkalemia generally resolves without intervention. It should be noted that 9.7% of patients treated with finerenone in FINEARTS-HF were reported to have hyperkalemia (vs 4.2% with placebo); however, the rate of hospitalization due to hyperkalemia was low (0.5% with finerenone vs 0.2% with placebo).^40^^,^^82^ These data suggest that selection of an appropriate starting dose of finerenone based on kidney function and dose adjustments due to regular potassium monitoring may mitigate the risk of developing a serious hyperkalemia event in clinical practice.^82^

#### Implementation of sMRAs in patients with HFrEF at risk of hyperkalemia and/or impaired kidney function

During treatment, serum potassium and eGFR levels need to be monitored, especially following changes in dose, and more frequent monitoring may be needed in patients with impaired kidney function. For spironolactone, serum potassium levels should be measured within 1 week of initiation or titration and regularly thereafter (Figures 1B and 3). If hyperkalemia occurs, the dose of spironolactone should be decreased or discontinued while the hyperkalemia is treated (Figures 1B and 3).^41^ More specific information regarding monitoring is provided in the product label for eplerenone. Serum potassium should be evaluated within the first week and 1 month after the start of treatment or dose adjustment (Figures 1D and 3) and should be assessed periodically thereafter.^43^ If serum potassium levels rise to 5.5 to 5.9 mEq/L, the dose of eplerenone should be reduced (from 50 mg once daily to 25 mg once daily, 25 mg once daily to 25 mg every other day, or 25 mg every other day to withhold treatment). Moreover, eplerenone should be withheld if serum potassium is ≥6.0 mEq/L, but treatment can be restarted when potassium levels fall below 5.5 mEq/L (Figure 1D).

Spironolactone and eplerenone both report a safety warning about hyperkalemia.^41^^,^^43^ Spironolactone is also associated with hypotension and worsening kidney function due to excessive diuresis, which may also cause symptomatic dehydration. Caution therefore needs to be applied when administering spironolactone to patients with HFmrEF/HFpEF, especially in light of the safety concerns observed with spironolactone compared with placebo in the SPIRIT-HF trial.^58^^,^^59^ Volume status and renal function should be measured periodically during treatment with spironolactone^41^ to monitor for these adverse effects. Electrolyte and metabolic abnormalities can also occur with spironolactone, including hyponatremia, hypomagnesemia, hypocalcemia, hypochloremic alkalosis, and hyperglycemia, so serum electrolytes, uric acid, and blood glucose should be monitored periodically. Spironolactone can also cause gynecomastia, which was observed in 9% of male patients with HFrEF in the RALES trial.^41^ However, symptoms usually resolve following treatment discontinuation^83^^,^^84^ but may persist in some instances. A high rate of discontinuation of spironolactone was also reported in SPIRIT-HF, which may have been related to side effects of the drug^58^; hence, clinicians need to remain vigilant when administering this medication to patients with HFmrEF/HFpEF.

#### Implementation of nsMRAs in patients with HFmrEF/HFpEF at risk of hyperkalemia and/or impaired kidney function

For finerenone, serum potassium and eGFR levels should be measured after 4 weeks following treatment initiation, and the dose of finerenone should be adjusted accordingly (Figure 1F).^22^ The dose-adjustment schedule depends on the primary indication for treatment (HFmrEF/HFpEF or CKD with T2D [Table 3 and Figure 2] and potassium levels [Figure 1F]). Serum potassium and eGFR levels should also be monitored after 4 weeks of finerenone treatment following a dose adjustment (Figures 1F and 2).^22^ In general, serum potassium and eGFR levels should be assessed periodically during treatment, but more frequent monitoring may be necessary in patients who are at risk of hyperkalemia.^22^

The label for finerenone includes a safety warning about hyperkalemia, and finerenone can also cause worsening of kidney function in patients with HFmrEF/HFpEF. Delaying uptitration or interrupting treatment with finerenone should be considered in patients with HFmrEF/HFpEF who develop clinically significant worsening of kidney function. To check for changes in kidney function, eGFR should be measured during initiation and titration as outlined and periodically in patients with HFmrEF/HFpEF.^22^ However, it is important to note that many therapies recommended and approved for HF, including finerenone, can cause an immediate reduction in eGFR, which is reversible and is most commonly due to a decrease in hemodynamic pressures in the glomeruli from the medication initiated rather than a true change in kidney function.^85^^,^^86^ It is therefore recommended to monitor the change in eGFR over a long period (eg, 3 years) to establish whether an initial dip in eGFR is an acute, reversible effect occurring as a result of medication initiation or a more serious issue.^85^ Indeed, a prespecified analysis from FINEARTS-HF (N = 5,587) and an exploratory analysis from FIDELITY (a prespecified, pooled individual-level analysis of the FIDELIO-DKD and FIGARO-DKD phase 3 trial data) in patients with CKD and T2D (N = 12,798) have found that the long-term benefits of finerenone are not modified by an acute eGFR decline observed within the first month of treatment.^86^^,^^87^

## sMRAs and nsMRAs in the wider HFmrEF and HFpEF treatment landscape

As outlined by the HFSA/ASPC scientific statement, secondary prevention of HF is needed to reduce the impact of the disease among patients with an established diagnosis of HF.^17^ Four pillars of therapy are now well established in HFrEF, with simultaneous treatment offering the greatest benefit, so it is reasonable to wonder whether a similar approach should be considered for HFmrEF/HFpEF. Some evidence is currently available for the use of spironolactone or finerenone in combination with other therapies for HFmrEF/HFpEF, and ongoing trials with approved MRAs will increase the evidence base for the use of existing therapies. Trials are also ongoing for HF with investigational agents that target aldosterone. The efficacy of these new drugs is being assessed in combination with existing therapies and may lead to further improved outcomes for HFmrEF/HFpEF; for example, balcinrenone plus dapagliflozin in the BalanceD-HF (Study to Evaluate the Effect of Balcinrenone/Dapagliflozin in Patients With Heart Failure and Impaired Kidney Function) study and vicadrostat plus empagliflozin in the EASi-HF (A Phase III Double-blind, Randomised, Parallel-group Superiority Trial to Evaluate Efficacy and Safety of the Combined Use of Oral Vicadrostat and Empagliflozin Compared With Placebo and Empagliflozin in Participants With Symptomatic Heart Failure and LVEF ≥40%) trial.^13^^,^^88^

The TOPCAT trial showed that spironolactone could be combined with other therapies for HFmrEF/HFpEF as the majority of patients were receiving concomitant treatment with ACEI/ARB or beta-blockers (N = 3,445; 84.2% and 77.8%, respectively).^54^ Furthermore, the SOGALDI-PEF (SOdium-Glucose cotransporter-2 inhibitor, ALDosterone-antagonIst, or both for heart failure with Preserved Ejection Fraction) study that was recently completed with a small number of patients with HFmrEF/HFpEF (N = 108) investigated combining spironolactone with the SGLT-2i dapagliflozin. The study found that N-terminal proBNP levels were reduced and hyperkalemia occurred less frequently with the combination vs dapagliflozin alone.^89^

Finerenone can also be combined with other therapies for HF. In ARTS-HF (N = 1,066), the majority of patients were already receiving treatment with ACEI/ARB or beta-blockers at baseline (78.1% and 86.0%, respectively), and a benefit with finerenone was observed vs eplerenone for HFrEF.^75^ Similarly, in FINEARTS-HF (N = 6,001), most participants with HFmrEF/HFpEF were being treated with finerenone on top of beta-blockers (84.9%) and an ARNi/ACEI/ARB (79.4%),^40^ and a further 13.6% of participants were receiving an SGLT-2i at baseline.^32^ In combination with this usual care, finerenone significantly reduced the primary outcome (a composite of total worsening HF events and death from CV causes) in HFmrEF/HFpEF vs placebo,^40^ and a prespecified subgroup analysis showed that the benefit of finerenone was consistent irrespective of ARNi/ACEI/ARB use and SGLT-2i use at baseline.^40^ However, it should be noted that the proportion of patients who received concomitant SGLT-2i was low (13.6%), and the subgroup analysis was insufficiently powered to assess the efficacy of the combination.^32^ Conclusive evidence for the efficacy of combined therapy with finerenone and an SGLT-2i in HF is therefore currently limited, especially given that SGLT-2is have only been recommended as a GDMT since 2021–2022,^1^^,^^2^^,^^4^^,^^90^ and finerenone was just approved by the FDA for the treatment of HFmrEF/HFpEF in July 2025.^22^ In the absence of definitive head-to-head trial data for HFmrEF/HFpEF, an actuarial analysis using currently available cross-trial data has assessed the potential effects of a combination therapy in this population.^91^ Patient-level data from trials of the SGLT-2i dapagliflozin (DELIVER, N = 6,263), finerenone (FINEARTS-HF, N = 6,001), and the ARNi sacubitril/valsartan (PARAGON-HF [Prospective Comparison of ARNI with ARB Global Outcomes in HF With Preserved Ejection Fraction], N = 4,796) were analyzed using established methods of indirect comparison. A combination therapy with finerenone and an SGLT-2i was estimated to reduce the risk of a composite of CV death and worsening HF by 31% in the overall population (Figure 4). In patients with HFmrEF/HFpEF and LVEF <60%, addition of an ARNi to an SGLT-2i and finerenone led to an estimated RRR of 39% for this same outcome.^91^ Long-term projections suggested that a combination therapy with finerenone and an SGLT-2i would provide additional CV-death- or HF-event-free 3.6 years compared with standard therapy in a patient aged 65 years with HFmrEF/HFpEF. This benefit was extended to 4.9 years in patients with HFmrEF/HFpEF and LVEF <60% when an ARNi was added to the treatment with finerenone and an SGLT-2i. It should be noted that these data have yet to be confirmed in a prospective head-to-head trial, and findings from this actuarial analysis may be confounded by several factors, including variable patient populations and background therapies used in the individual trials, potential for nonadditive effects of the different agents, and assumption of multiplicative effects without a formal statistical analysis. However, the potential for substantial benefits with event-free survival supports the idea of implementation of early comprehensive treatment in all patients with HFmrEF/HFpEF.^91^ Definitive evidence for the efficacy of a combination therapy with finerenone and an SGLT-2i will be available in time from the ongoing CONFIRMATION-HF trial (N = 1,500), which is assessing the combination therapy with finerenone and empagliflozin in patients hospitalized for HF.^37^^,^^71^

**Figure 4:**
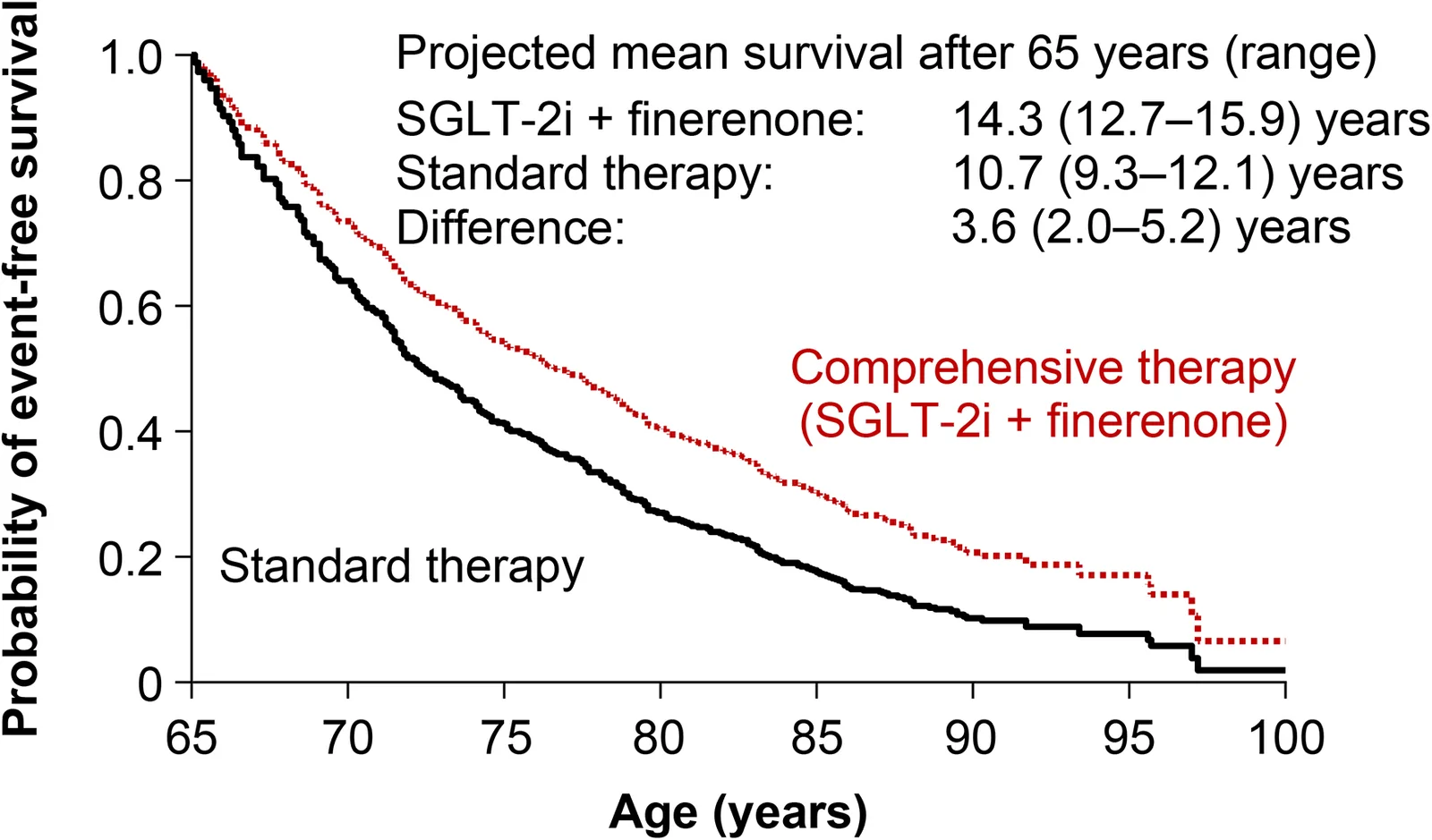
Estimated Absolute Event-Free Survival Gains in an Actuarial Analysis of HF With Mildly Reduced or Preserved Ejection Fraction With Comprehensive Medical Therapy of an SGLT-2i and Finerenone Data are from an indirect comparison of cross-trial data from DELIVER, FINEARTS-HF, and PARAGON-HF trials (N = 6,263, N = 6,001, and N = 4,796, respectively). Kaplan-Meier estimated curve for patients starting at 65 years of age for survival free from the primary endpoint (cardiovascular death or worsening HF event) in the overall HF with mildly reduced or preserved ejection fraction population. SGLT-2i = sodium–glucose cotransporter-2 inhibitor.

Reprinted from Vaduganathan et al. *Nat Med*. 2026;32:325-331. with permission. Copyright 2025 The Authors. http://creativecommons.org/licenses/by/4.0/.^91^

Outside of HF, supportive evidence for concomitant treatment with finerenone and an SGLT-2i in cases of HFmrEF/HFpEF has come from the CONFIDENCE (Combination Effect of Finerenone and Empagliflozin in Participants with Chronic Kidney Disease and Type 2 Diabetes Using a Urinary Albumin-to-Creatinine Ratio Endpoint) trial in patients with CKD and T2D. The CONFIDENCE trial demonstrated a greater reduction in urine albumin-to-creatinine ratio, a marker of kidney disease, with the finerenone and empagliflozin combination therapy vs monotherapy with either agent (N = 818; *P* < 0.001).^92^ Findings from this trial also indicated a possible relative reduction in the incidence of hyperkalemia with the combination therapy in patients with CKD and T2D. The frequency of hyperkalemia events was 15% to 20% lower with finerenone and empagliflozin vs finerenone alone (9.3% vs 11.4%, respectively). As expected, the frequency of hyperkalemia events was lowest with empagliflozin monotherapy (3.8%).^92^ Safety data from the ongoing CONFIRMATION-HF trial will provide insights into hyperkalemic events in patients with HF.^37^^,^^71^

Based on the available evidence demonstrating significant clinical benefits in patients with HFmrEF/HFpEF, SGLT-2is and finerenone are considered as foundational therapies and are approved by the FDA for use in these patients (Figure 5).^29^^,^^73^^,^^93^, ^94^, ^95^ SGLT-2is are strongly recommended for all patients with HFmrEF/HFpEF based on clinical evidence from the EMPEROR-preserved (EMPagliflozin outcomE tRial in Patients With chrOnic heaRt Failure With Preserved Ejection Fraction) and DELIVER (Dapagliflozin Evaluation to Improve the LIVEs of Patients With PReserved Ejection Fraction Heart Failure) trials^4^ that demonstrated a modest absolute reduction in risk of HHF or CV death (∼3% for both agents and number needed to treat of ∼30).^96^ Universal use of finerenone is still debated however, owing to a modest overall absolute reduction in the risk of worsening HF or CV death vs placebo in FINEARTS-HF (3.2% and number needed to treat of 30-35)^96^ and data from some prespecified subgroup analyses of FINEARTS-HF that may indicate variability of response to finerenone among patients.^97^, ^98^, ^99^ Despite the primary endpoint being reduced irrespective of baseline NYHA class (*P*_interaction_ = 0.54), greater absolute benefits may be observed in patients with higher symptom severity (absolute rate reduction of 4.5 events vs 2.0 events per 100 patient-years for NYHA class III/IV vs NYHA class II, respectively).^97^ Similarly, although the primary endpoint was reduced irrespective of the recency of a worsening HF event (*P* = 0.07), those who had experienced an event within 7 days of baseline may have a greater risk reduction for the primary endpoint with finerenone than patients with an event >3 months from baseline or no prior event (risk ratio of 0.74 [95% CI: 0.57-0.95] and 0.99 [95% CI: 0.81-1.21], respectively).^98^ Finally, the benefit of finerenone was consistent across the ejection fraction spectrum for HFmrEF/HFpEF (*P*_interaction_ = 0.70); however, it should be noted that the confidence intervals were wider for event outcomes in patients with higher LVEFs (rate ratio of 0.84 [95% CI: 0.68-1.03], 0.80 [95% CI: 0.66-0.97], and 0.94 [95% CI: 0.70-1.25] for LVEF <50%, ≥50% to <60%, and ≥60%, respectively).^99^ It is unclear whether this was due to the low number of reported events, which limited statistical power,^99^ or a possible lack of or reduced benefit with finerenone in patients with higher LVEFs akin to the results observed with spironolactone in the TOPCAT subanalysis according to LVEF.^55^ Other drug classes that may be beneficial in select patients with HFmrEF/HFpEF include ARNi, beta-blockers, and glucagon-like peptide-1 receptor (GLP-1R) agonists. The ARNi sacubitril/valsartan is FDA approved for use in HFmrEF/HFpEF for patients who have an LVEF below normal (<60%).^19^^,^^21^ However, current guidelines from AHA/ACC/HFSA include a class IIb recommendation for ARNis, ACEI, and ARBs in HFmrEF/HFpEF given the primary outcome of the PARAGON-HF trial was not met and other analyses are exploratory.^2^^,^^20^ The AHA/ACC/HFSA guidelines also include class IIb recommendations for beta-blockers (that have demonstrated benefit in HFrEF) in HFmrEF, but these are not recommended for HFpEF.^2^ While emerging data suggest stimulation of the GLP-1R may reduce the risk of CV death or worsening HF in patients with obesity and HFpEF, GLP-1R agonists are not yet approved for this indication or recommended in the current AHA/ACC/HFSA guidelines for HF.^2^^,^^93^^,^^94^

**Figure 5:**
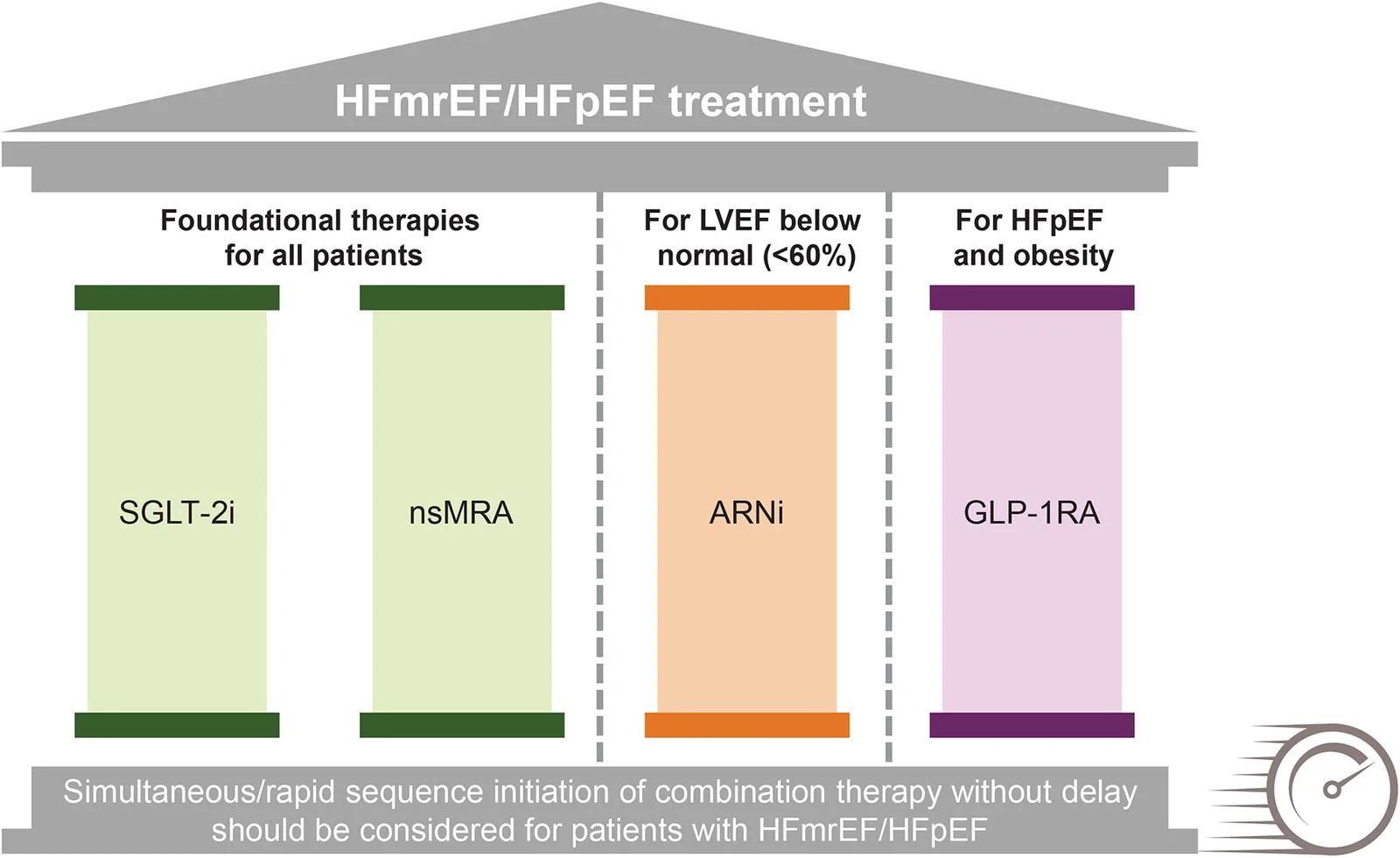
Pillars of Treatment for HFmrEF/HFpEF^29^^,^^93^, ^94^, ^95^ ARNi = angiotensin receptor–neprilysin inhibitor; GLP-1RA = glucagon-like peptide-1 receptor agonist; HFmrEF/HFpEF = heart failure with mildly reduced or preserved ejection fraction; nsMRA = nonsteroidal mineralocorticoid receptor antagonist; other abbreviations as in Figures 1 and 4.

### Treatment of HFmrEF/HFpEF with comorbid CKD

HF is now increasingly considered part of an inter-related set of CKM disorders that overlap (Figure 6),^100^, ^101^, ^102^ with newer therapies being able to effectively target multiple aspects of CKM diseases.^100^ CKD, often in combination with T2D, is one such disorder that is commonly found in individuals with HF. Available evidence shows that CKD and HF share similar pathophysiological pathways, disease drivers, and risk factors for development and progression.^103^ As such, there is significant overlap in treatment options for HF and CKD in patients with T2D, with ACEI/ARB, SGLT-2is, and finerenone all being recommended by Kidney Disease: Improving Global Outcomes for the management of people with CKD and T2D,^67^ and these agents are also all included in a proposed framework for HF prevention through CKM-oriented interventions.^100^ In line with this, the focused update of ESC HF guidelines in 2023 includes a class I recommendation for finerenone to reduce the risk of HHF in people with CKD and T2D based on data from FIDELIO-DKD and FIGARO-DKD.^4^ Newer guidelines from iCARDIO Alliance and JCS/JHFS published in 2025 include similar recommendations for finerenone use in this patient population (Table 2).^10^^,^^62^

**Figure 6:**
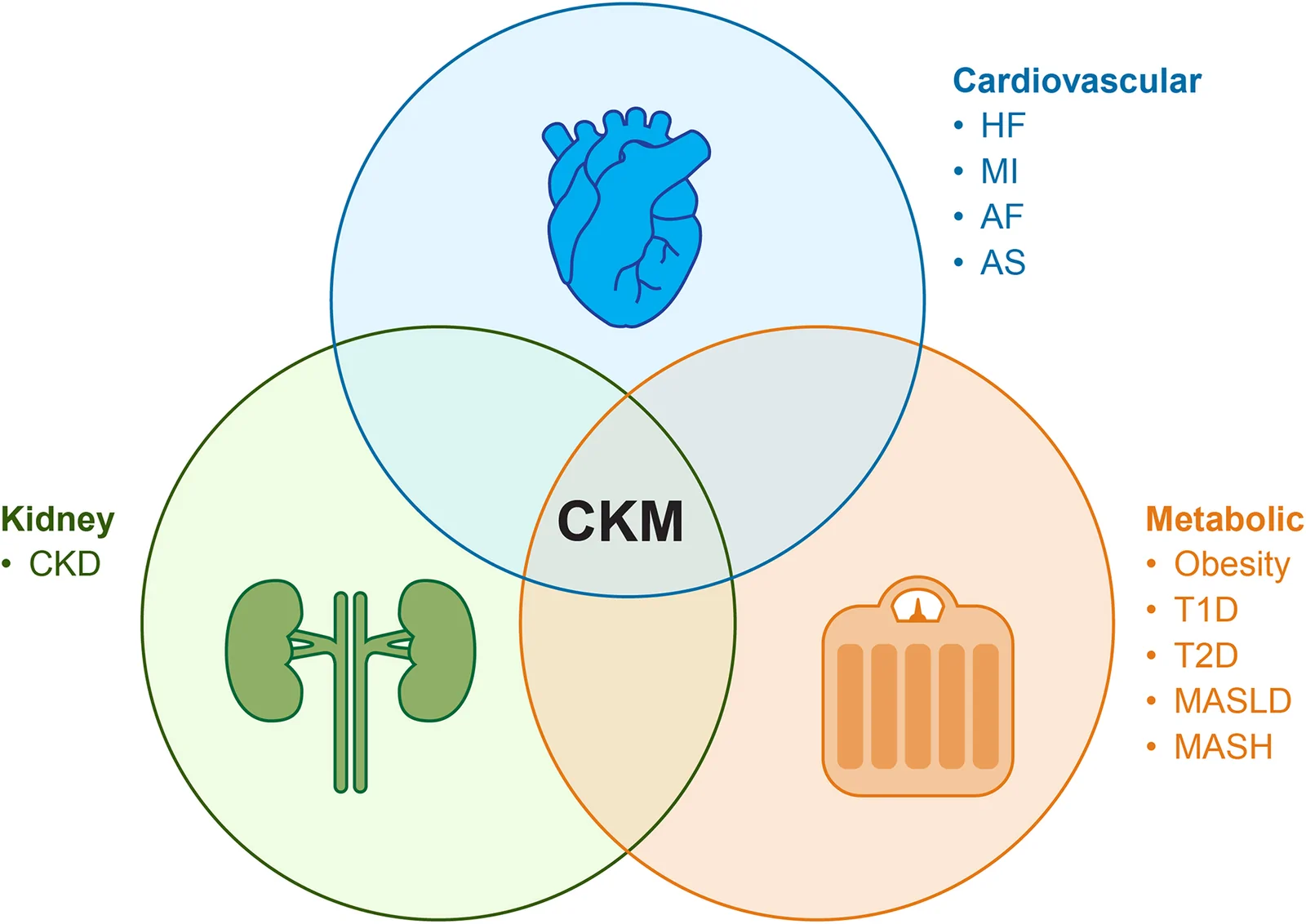
Overlapping Disorders in CKM Syndrome^100^, ^101^, ^102^ AF = atrial fibrillation; AS = aortic stenosis; CKD = chronic kidney disease; CKM = cardiovascular–kidney–metabolic; HF = heart failure; MASH = metabolic dysfunction-associated steatohepatitis; MASLD = metabolic dysfunction-associated steatotic liver disease; MI = myocardial infarction; T1D = type 1 diabetes; T2D = type 2 diabetes.

Epidemiology data reveal that around 50% of patients with HF have some form of kidney impairment.^103^ Data from the Swedish Heart Failure Registry (N = 40,230) showed that prevalence of CKD is highest in HFpEF cases compared with HFmrEF or HFrEF cases (56% vs 48% vs 45%, respectively; *P* < 0.001).^104^ Other observational cohorts and a contemporary patient population suggest that CKD is present in up to 49% and 62% of patients with HFpEF, respectively.^105^ As these conditions often occur together, it can be challenging for health care professionals (HCPs) to identify which is the primary disease.^103^ It is therefore important for HCPs to consider appropriate management of patients with both HF and CKD.

There are some key differences between the indications for the use of finerenone in cases of HFmrEF/HFpEF and CKD with T2D that need to be taken into account before the initiation of treatment, such as the target dose for treatment, the dose-adjustment protocol according to serum potassium levels, and a specific warning for worsening kidney function in patients with HFmrEF/HFpEF (Table 3).^22^ This means that HCPs who are treating patients with both CKD and HFmrEF/HFpEF need to consider the following practical points. First, dosing of finerenone should reflect the primary indication being treated (Figure 2). In the absence of a specific label or guideline direction on dose selection when both indications coexist, we suggest that for patients who meet the HFmrEF/HFpEF indication, the HF indication should be regarded as the primary indication. Finerenone should therefore be titrated toward the HF target dose (based on kidney function at initiation) as tolerated (Figure 1E). This dose range was shown to be efficacious and safe in FINEARTS-HF, including in patients with concomitant CKD, and preserves the CV benefits established for finerenone in CKD with T2D.^40^^,^^69^ The CKD and T2D dosing scheme applies when CKD with T2D is the sole qualifying indication (Figure 1E). Note that the target dose for the specific indication should remain the same once treatment has begun as the target dose is determined by the eGFR level at treatment initiation.^22^ If a decrease in eGFR of >30% is observed during titration to the target dose in either indication, then the current dose of finerenone should be maintained until the decrease in eGFR is ≤30% compared with the previous measurement.^22^ It should be noted that differences in dosing recommendations between indications are based on finerenone trial designs for HFmrEF/HFpEF and CKD with T2D rather than any safety concerns. Second, as mentioned earlier, HCPs need to be aware of the possibility of a short-term drop in eGFR with GDMT for HF including finerenone, SGLT-2i, and spironolactone due to the initiation of the medication rather than an adverse effect on the kidney.^85^, ^86^, ^87^^,^^106^^,^^107^ A possible acute decline in eGFR should not influence the decision to begin treatment with finerenone, provided kidney function is above the threshold for initiation (eGFR ≥25 mL/min/1.73 m^2^), and it should not automatically lead to treatment discontinuation if observed after initiation. Patients should be monitored over a long period (eg, 3 years) to establish whether an initial dip in eGFR is truly a genuine decline in kidney function.^85^ Finally, once finerenone has been initiated, treatment can be continued until a patient has progressed to end-stage kidney disease (eGFR <15 mL/min/1.73 m^2^) as long as potassium levels remain within the required limit.^46^

The benefits of a particular agent across the CKM spectrum should be considered when selecting appropriate treatment for patients with HFmrEF/HFpEF who have comorbidities such as CKD. Finerenone is already approved for CKD with T2D, with a demonstrated reduction in adverse kidney and CV outcomes over time.^22^ Importantly, the CV benefits observed in patients with CKD and T2D were largely driven by a strong reduction in HHF (RRR of 22% vs placebo; *P* = 0.0030) in the FIDELITY population that excluded HFrEF before randomization and had a low level of established HF at baseline (7.7%).^108^ In line with consideration of appropriate treatment for patients with HF and comorbidities, a strategic imperative was issued in 2022 to focus on the need for a dedicated cardiorenal multidisciplinary team to manage the treatment of patients with coexisting HF and CKD.^109^ Development of educational programs that span all levels of cardiology and nephrology will help train HCPs to diagnose, treat, and prevent adverse outcomes in patients with all LVEF classes of HF and CKD. Furthermore, a presidential advisory from the AHA has highlighted the critical need for clarity on the definition of CKM disease, the staging strategies that promote life-long prevention of disease, the use of predictive algorithms that include the most relevant exposures and outcomes, and the optimized management of disease across a range of clinical specialties to improve CKM health and subsequent outcomes.^102^

## Conclusions

MRAs are a fundamental treatment option for the management of HF, with demonstrated efficacy across the entire LVEF spectrum when considered as a single therapeutic class. FDA approvals vary between the classes of MRA, with the sMRAs, spironolactone and eplerenone, being indicated for HFrEF, and the nsMRA finerenone being approved for HFmrEF/HFpEF based on data from the recent FINEARTS-HF study. Guidelines strongly recommend sMRA for the treatment of HFrEF, but the evidence base for HFmrEF/HFpEF is currently more limited, thereby resulting in weaker recommendations for this patient population. However, stronger recommendations are now emerging for the specific use of finerenone in HFmrEF/HFpEF, and ongoing trials may improve the strength of the evidence in HF still further.

This review serves as a call to action for clinicians to increase the use of GDMT for HF, with a particular focus on patients with HFmrEF/HFpEF. Although the benefits of combination treatment in HFrEF are well established, GDMT is often underutilized and remains an unmet need. Rapid initiation of a quadruple therapy with the 4 pillars of treatment, which include the sMRAs spironolactone and eplerenone, is strongly recommended and is extremely important to reduce clinical risk in HFrEF. Akin to schedules already recommended for patients with HFrEF, we propose herein a similar pillar approach for the treatment of HFmrEF/HFpEF that includes simultaneous or rapid sequence initiation of foundational therapies, SGLT-2i and finerenone, without delay in all patients. Management of HFmrEF/HFpEF also needs to be evaluated in conjunction with other CKM disorders to ensure that treatment is optimized and individualized to target multiple aspects of the disease. In addition to being approved for use in HFmrEF/HFpEF, finerenone is also indicated for CKD with T2D, which are disorders commonly found in patients with HF. The apparent clinical benefits of the nsMRA finerenone in HFmrEF/HFpEF should encourage the initiation of treatment without delay following a diagnosis, in combination with other therapies that have proven efficacy in this patient population.

## Funding support and author disclosures

This review was sponsored by Bayer AG. Medical writing support was provided by Kate Crawford (Omnicom Health Medical Communications) and funded by Bayer AG. The views and opinions expressed in this article are those of the authors. Bayer AG reviewed the article for medical accuracy but did not provide input on or influence the authors’ scientific judgments or conclusions. Dr Michos is on the consulting/advisory boards of the following companies: Arrowhead, Bayer, Boehringer Ingelheim, Edwards Life Science, Eli Lilly, Ionis, Merck, New Amsterdam, Novartis, Novo Nordisk, and Pfizer. Dr Ambrosy has received research support through grants from the National Institutes of Health (R01HL173866 and R01AG091005), the Food and Drug Administration (U01FD008704), The American Heart Association, The Permanente Medical Group, Kaiser Permanente Northern California Community Benefits Programs, Garfield Memorial Fund, Abbott Laboratories, Abiomed, Amarin Pharma, Inc, Bayer Pharma AG, Boehringer Ingelheim, Cordio Medical, Edwards Lifesciences LLC, Esperion Therapeutics, Inc., Merck, and Novartis; and has consulted for Bristol Meyer Squibb, Bayer Pharma AG, Corstasis, Merck, Novo Nordisk, Pharmacosmos A/S, Pfizer, scPharma, SQ Innovation, Reprieve Cardiovascular, and VisCardia. Dr Fonarow has served as a consultant for Abbott, Amgen, AstraZeneca, Bayer, Boehringer Ingelheim, Cytokinetics, Eli Lilly, Johnson & Johnson, Medtronic, Merck & Co, Inc, Novartis, and Pfizer.
